# Improving MetFrag with statistical learning of fragment annotations

**DOI:** 10.1186/s12859-019-2954-7

**Published:** 2019-07-05

**Authors:** Christoph Ruttkies, Steffen Neumann, Stefan Posch

**Affiliations:** 1 Department Biochemistry of Plant Interactions, Leibniz Institute of Plant Biochemistry, Weinberg 3, Halle (Saale), 06120 Germany; 2 German Centre for Integrative Biodiversity Research (iDiv) Halle-Jena-Leipzig, Deutscher Platz 5e, Leipzig, 04103 Germany; 3 Institute of Computer Science, Martin Luther University Halle-Wittenberg, Von-Seckendorff-Platz 1, Halle (Saale), 06099 Germany

**Keywords:** Mass spectrometry, Statistical modeling, Identification

## Abstract

**Background:**

Molecule identification is a crucial step in metabolomics and environmental sciences. Besides in silico fragmentation, as performed by MetFrag, also machine learning and statistical methods evolved, showing an improvement in molecule annotation based on MS/MS data. In this work we present a new statistical scoring method where annotations of *m/z* fragment peaks to fragment-structures are learned in a training step. Based on a Bayesian model, two additional scoring terms are integrated into the new MetFrag2.4.5 and evaluated on the test data set of the CASMI 2016 contest.

**Results:**

The results on the 87 MS/MS spectra from positive and negative mode show a substantial improvement of the results compared to submissions made by the former MetFrag approach. Top1 rankings increased from 5 to 21 and Top10 rankings from 39 to 55 both showing higher values than for CSI:IOKR, the winner of the CASMI 2016 contest. For the negative mode spectra, MetFrag’s statistical scoring outperforms all other participants which submitted results for this type of spectra.

**Conclusions:**

This study shows how statistical learning can improve molecular structure identification based on MS/MS data compared on the same method using combinatorial in silico fragmentation only. MetFrag2.4.5 shows especially in negative mode a better performance compared to the other participating approaches.

**Electronic supplementary material:**

The online version of this article (10.1186/s12859-019-2954-7) contains supplementary material, which is available to authorized users.

## Background

The identification of small molecules such as metabolites is a crucial step in metabolomics and environmental sciences. The analytical tool of choice to achieve this goal is mass spectrometry (MS) where ionized molecules can be differentiated by their mass-to-charge (*m/z*) ratio. As a single *m/z* value is not sufficient for the unequivocal determination of the molecular structure, tandem mass spectrometry (MS/MS) is applied, which results in the formation of fragment ions of the entire molecule. These fragments result in fragment peaks that are characterized by their *m/z* and intensity value. The intensity correlates with the amount of ions detected with that particular *m/z* value. These *m/z* fragment peaks can be used to infer additional hints about the underlying molecular structure.

The interpretation of the generated data is complex and usually requires expert knowledge. Over the past years, several software tools have been developed to overcome the time-consuming manual analysis of the growing amount of MS/MS spectra in an automated way. The first approaches tried to reconstruct observed fragment spectra by performing in silico fragmentation in either a rule based (e.g. MassFrontier [1]) or combinatorial manner such as MetFrag [2, 3], MIDAS [4], MS-Finder [5] and MAGMa [6].

MetFrag was one of the first combinatorial approaches developed and performs in silico fragmentation of molecular structures. Given a single MS/MS spectrum of an unknown molecule, MetFrag first selects molecular candidates from databases given the neutral mass of the parent ion. In the next step, each of the retrieved candidates is treated individually and fragmented in silico using a bond-disconnection approach. The generated fragment-structures are assigned to the *m/z* fragment peaks of the MS/MS spectrum, based on the comparison of the theoretical mass of the generated structure and the *m/z* value of the acquired fragment peak. Given a set of assignments of *m/z* fragment peaks to fragment-structures for one candidate, MetFrag calculates a score that indicates how well the candidate matches the given MS/MS spectrum. These scores are used to rank all retrieved candidates. Ideally, the correct one is ranked in first place.

Statistical approaches have evolved, which are learning fragmentation processes on the basis of annotated experimental MS/MS data. CFM-ID [7] is using Markov-chains to model transitions of fragment-structures for the prediction of MS/MS spectra. Generated spectra can be aligned with the spectrum of interest and report the candidates with the best matching spectral prediction. FingerID [8] uses MS/MS spectra to predict molecular fingerprints. These Fingerprints are bit-wise representations of molecular structures where each position in the fingerprint encodes a structural property of the underlying molecule. FingerID uses support vector machines (SVM) and is enhanced by CSI:FingerID (CSI:FID) [9], integrating fragmentation trees which are calculated by SIRIUS [10]. CSI:IOKR [11] replaces the SVM prediction by an input-output kernel regression approach. Recent analysis in one of the latest CASMI (Critical Assessment of Small Molecule Identification) contests (2016) [12] reveal that techniques supported by statistical learning (i.e. CSI:FID and CSI:IOKR) are the most promising and powerful methods used to perform structure elucidation if only the MS/MS data is considered.

In this work we introduce a new statistical approach to evaluate candidates for MS/MS spectra. Using training data, probabilities of the predicted fragment-structures given the observed *m/z* peaks are estimated with a Bayesian approach. These probabilities are integrated as new scoring terms for MetFrag to rank candidates. The new scoring schema is tested on the challenge data sets of the CASMI contest 2016. The method shown here complements the different machine learning and statistical approches that perform MS/MS spectra prediction (CFM-ID), prediction of molecular fingerprints (CSI:FID, CSI:IOKR) and now combining in silico fragmentation and statistical scoring for the evaluation of retrieved molecular candidates. The new scoring functions are available with the new MetFrag version 2.4.5.

## Methods

This section introduces the notation and the Bayesian model approach used to evaluate how likely a fragment-structure is in the presence of an *m/z* fragment peak. The resulting probabilities are defined across the domain of all possible fragment-structures and all *m/z* fragment peaks, but can be reduced to become tractable. The resulting probability distribution will be used in the candidate score $S^{c}_{RawPeak}$ indicating whether a candidate can explain the *m/z* fragment peaks with fragment-structures seen in the training spectra. In analogy, neutral losses will also be considered. The parameter estimation to model the probability distribution is at the heart of our approach. We describe how they are estimated from training data, taking care to clearly separate training data from evaluation data. Finally we describe the evaluation using the CASMI 2016 challenge data and comparison to the results obtained by other approaches and state-of-the art small molecule identification programs.

First, we introduce notations required for our approach. A summary of the notation used in the following and their description can be found in Additional files 4 and 5: Tables S1 and S2. Consider a set of *N* centroided MS/MS spectra $\underline {m}=\{\underline {m}_{n}|n=1,\dots N\}$ where $\underline {m}_{n} = (m_{n1},\dots m_{n{K_{n}}})$ consists of *K*_*n*_*m/z* fragment peaks *m*_*nk*_. Furthermore, for each spectrum $\underline {m}_{n}$ a set of candidates $\underline {c}_{n}$ of length *C*_*n*_ is given, typically retrieved from a database. For a given candidate $c_{nc} \in \underline {c}_{n}$, MetFrag performs an in silico fragmentation and assigns each observed *m/z* fragment peak *m*_*nk*_ to one of the generated fragment-structures, denoted *f*_*nck*_ in the following. This can be interpreted as explaining the *m/z* fragment peak *m*_*nk*_ with the fragment-structure *f*_*nkc*_. On the basis of the in silico fragmentation, assignments of *m/z* fragment peaks to fragment-structures $(\underline {m}_{n}, \underline {\smash {f}}_{nc}), c=1,\dots C_{n}$, are determined. As there is not necessarily a matching fragment-structure for every *m/z* fragment peak *m*_*nk*_, we introduce ⊥ in case an *m/z* fragment peak *m*_*nk*_ cannot be annotated, and denote *f*_*nck*_=⊥ in this case.

As stated in the introduction, we want to evaluate candidates for an MS/MS spectrum by a statistical scoring approach to be integrated into MetFrag. Therefore, we apply a scoring term based on the probability $P(\underline {\smash {f}}_{nc} | \underline {m}_{n})$. The distribution $P(\underline {\smash {f}} | \underline {m})$ models the occurence of fragment-structures in $\underline {\smash {f}}$ in the correct candidate for a given list $\underline {m}$ of *m/z* fragment peaks in an observed spectrum. In the following we assume the independence of the assignments of *m/z* fragment peaks to fragment-structures yielding

$$P(\underline{\smash{f}} | \underline{m}) = \prod_{k=1}^{K} P(f_{k} | m_{k}), $$

with $\underline {m} = (m_{1},,\dots,m_{K})$ and $\underline {\smash {f}} = (f_{1},\dots f_{K})$. From a chemical point of view, we know that certain *m/z* fragment peaks occur concurrently with other *m/z* fragment peaks (or at least with a higher certainty) due to multi-stage fragmentation pathways that lead to a further fragmentation of a generated fragment-structure. However, for the sake of model simplification we do not consider this information when assuming independence of assignments of *m/z* fragment peaks to fragment-structures.

A fragment-structure can be regarded as a connected charged molecular structure consisting of atoms connected via bonds. A graph can be used as data structure to represent a fragment-structure, as atoms and bonds can be represented by graph nodes and edges, respectively. However, to reduce the computational costs for comparing graphs by determining graph isomorphisms, especially when working with thousands or even hundreds of thousands of fragment-structures, we use molecular fingerprints as a bit-string representation of a molecular structure. Each bit of the fingerprint describes the presence or absence of a molecular feature within the structure. As different fragment-structures may share the same fingerprint, this approach reduces the the domain size and also generalizes very similar fragment-structures that would explain the same *m/z* fragment peak. There are different molecular fingerprint functions available, e.g., the MACCSFingerPrint [13] and the LingoFingerprint [14]. A fragment-structure fingerprint is defined as $\widetilde {f}_{k} = MolFing(f_{k})$, calculated by the fingerprint function *MolFing*.

We regard two fragment-structures *f* and *f*^′^ to be equal, if $\widetilde {f}$ and $\widetilde {f'}$ are equal, although *f* and *f*^′^ might be structurally different. This reduces the comparison to constant time as the fingerprint length is independent of the size of the fragment-structure. The distribution can now be re-defined as

$$P(\underline{\smash{\widetilde{f}}} | \underline{m}) = \prod_{k=1}^{K} P(\widetilde{f}_{k} | m_{k}). $$

The comparison of two *m/z* fragment peaks *m* and *m*^′^ can not be performed as a simple test for equality by *m*=*m*^′^. This is impractical for MS measurements as they show a certain degree of deviation depending on the mass accuracy of the instrument. For this reason, the m/z range covered by training and test spectra is discretized into non-equidistant bins [*b*_*i*_,*b*_*i*+1_]. The boundaries are calculated as *b*_*i*+1_=*b*_*i*_+2·(*mzppm*(*b*_*i*_)+*mzabs*) with *b*_0_ set to the minimum mass value of this range. The values *mzabs* and *mzppm*(*b*_*i*_) represent the absolute (in *m/z*) and relative mass (in ppm) deviation given by the MS setup.

Two *m/z* fragment peaks *m* and *m*^′^ are considered to be equal if they fall into the same bin. In the following each *m/z* fragment peak *m* is discretized to the central value of its bin.

### Domains and Parameters

As a next step, the two domains *M* of *m/z* values *m* and *F* of all fragment-structure fingerprints $\widetilde {f}$ need to be defined. For *M* one could consider all bins resulting from discretization. However, this is impractical as the major part of this domain is not observed for a given data set. Likewise, the domain *F* can be defined to contain all possible fragment-structure fingerprints. Using the MACCSFingerprint with 166 bits would result in 2^166^≈9.35·10^49^ different fingerprints. In practice this space needs to be reduced to be tractable, and again only a fraction will be observed for a given problem. For a spectral training data set of *N* MS/MS spectra and *C*_*n*_ candidates each, we define a reduced peak domain $\widetilde {M}_{tr}$ and a reduced fingerprint domain $\widetilde {F}_{tr}$ as

$$\begin{array}{*{20}l} \widetilde{M}_{tr} &= \{m_{nk} | n \in 1,\dots N, k=1,\dots K_{n} \} \subseteq M \\ \widetilde{F}_{tr} &\,=\, \left\{\widetilde{f}_{nck} | n \!\in\! 1,\dots N, c\,=\,1,\dots C_{n}, k\,=\,1,\dots K_{n} \right\} \subseteq F, \end{array} $$

which are the *m/z* fragment peaks and fragment-structure fingerprints observed in this data set.

Furthermore, we define $\mathcal {D}_{train}$ as a list of all assignments of *m/z* fragment peaks to fragment-structures in the training data, i.e.

$${} \mathcal{D}_{train} \,=\, \left((m_{nk}, f_{nck}) | n \,=\, 1, \dots N, c\,=\,1,\dots C_{n}, k \,=\, 1, \dots K_{n} \right). $$

**Fig. 1:**
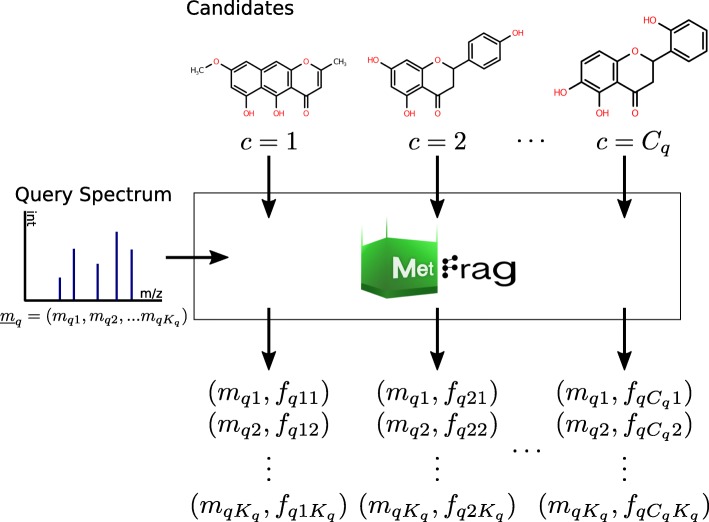
MetFrag processing of a single query spectrum ($\underline {m}_{q}$). The input for a MetFrag processing run is a query MS/MS spectrum and the candidate list. Fragments are generated in silico for each candidate and mapped to *m/z* fragment peaks in the given spectrum. The output is a list of assignments of *m/z* fragment peaks to fragment-structures for each candidate

Besides the MS/MS spectra given in this training data set we also need to address observations of an additional centroided MS/MS query spectrum $\underline {m}_{q}$ that is not part of the training data set. The processing of $\underline {m}_{q}$ is illustrated in Fig. 1. The domains are extended by the observations retrieved from this single query spectrum with *C*_*q*_ candidates and *K*_*q*_*m/z* fragment peaks, i.e.

$$\begin{array}{*{20}l} \widetilde{M} &= \widetilde{M}_{tr} \cup \{m_{qk} | k=1,\dots K_{q} \}\\ \widetilde{F} &= \widetilde{F}_{tr} \cup \{\widetilde{f}_{qck} | c=1,\dots C_{q}, k=1,\dots K_{q} \}. \end{array} $$

To define the distribution $P(\underline {\smash {\widetilde {f}}} | \underline {m})$ with $m \in \widetilde {M}$ and $\widetilde {f} \in \widetilde {M}$, we introduce the notation $\theta _{m\widetilde {f}} := P(\widetilde {f}|m)$, which is the probability of fragment-structure fingerprint $\widetilde {f}$ given an observed mass *m*. The complete set of parameters is given as

$$\underline{\theta} = (\theta_{m\widetilde{f}}), \quad\text{for}\quad m \in \widetilde{M}, \widetilde{f} \in \widetilde{F}. $$

### Parameter estimation

**Fig. 2:**
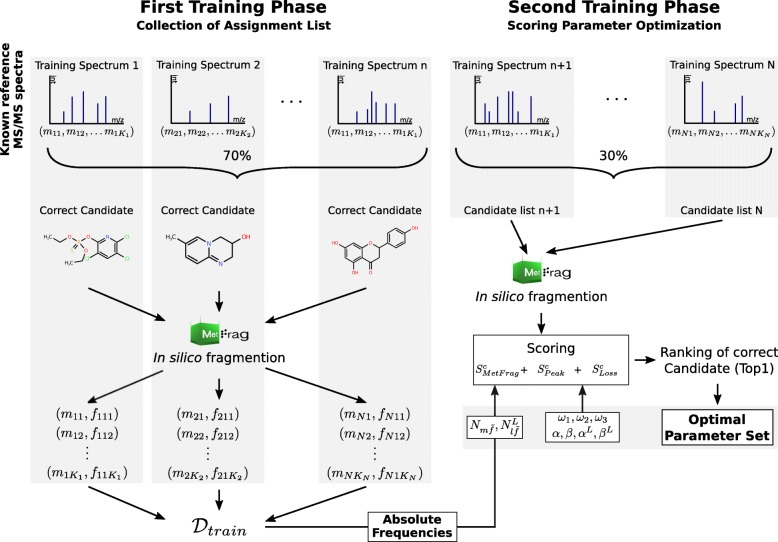
The training phase. The training consists of two major phases. For each phase a subset of the known reference MS/MS spectra is used. In the first phase MetFrag generates a list of assignments of *m/z* fragment peaks to fragment-structures for the given MS/MS spectra and their correct candidates. These assignments are generated by the in silico fragmentation of the correct candidate and the mapping of the generated fragment-structures to the *m/z* fragment peaks in the training spectrum. This assignments list ($\mathcal {D}_{train}$) is used in the second training phase along with the second subset of the reference spectra. Here, for each MS/MS spectrum the correct candidate is ranked with a candidate list using the consensus candidate score integrating besides the fragmenter ($S^{c}_{MetFrag}$) the two new statistical scoring terms ($S^{c}_{Peak}, S^{c}_{Loss}$). The number of correct Top1 rankings is used to optimize pseudo count and scoring weight parameters. The first training phase is used in analogy for the generation of the list containing assignments of *m/z* fragment losses to fragment-structures ($\mathcal {D}^{L}_{train}$)

The parameters are initially not known and need to be estimated from the training data. In the process of parameter estimation $\underline {c}_{n}$ is set to only contain the known correct candidate (*C*_*n*_=1) for the generation of $\mathcal {D}_{train}$ as this results in mainly correct predicted fragment-structure assignments as ground truth. The generation of $\mathcal {D}_{train}$ is illustrated in Fig. 2 where only the correct candidate for each spectrum is processed. One paradigm for parameter estimation is the maximum likelihood principle

$$ \underline{\hat{\theta}}^{ML} = \underset{\underline{\theta}}{\text{argmax}}~P(\mathcal{D}_{train}|\underline{\theta}), $$

which results in

$$\begin{aligned} &\hat{\theta}^{ML}_{m\widetilde{f}} = \frac{N_{m\widetilde{f}}}{{\sum\nolimits}_{\widetilde{f}' \in \widetilde{F}} ~N_{m\widetilde{f}'}},\\ & \quad \text{ with} \quad N_{m\widetilde{f}} = \sum\limits_{(m_{t},\widetilde{f_{t}}) \in \mathcal{D}_{train}} \delta(\widetilde{f}_{t},\widetilde{f})\delta(m_{t},m) \end{aligned} $$

$N_{m\widetilde {f}}$ is the absolute frequency of the assignments of *m/z* fragment peaks to fragment-structures $(m,\widetilde {f})$ in the training data set $\mathcal {D}_{train}$.

If such an assignment $(m, \widetilde {f})$ resulting from the query spectrum is not contained in the training data, a probability $\hat {\theta }^{ML}_{m\widetilde {f}} = 0$ is estimated. As a consequence the probability $P(\underline {\smash {\widetilde {f}}} | \underline {m})$ for the query will be zero.

Due to the limitation of the available training data, this situation will arise quite often. To avoid this problem, we use the Bayes paradigm including a priori distribution for the parameters to be estimated. In addition, as we only consider the correct candidate for each spectrum in $\mathcal {D}_{train}$ it is not possible to reliably estimate parameters in case $\widetilde {f} = \perp $, which is the probability for an *m/z* fragment peak without an assigned fragment-structure. Within the Bayesian approach we model this probability with the prior distribution and set *N*_*m*⊥_=0.

In the following we will use the mean posterior (MP) principle

$$\hat{\theta}_{m\widetilde{f}}^{MP} = E_{P(\underline\theta|\mathcal{D}_{train},\pi)}[\underline{\theta}] $$

where

$$P(\underline\theta|\mathcal{D}_{train},\pi) = \frac{P(\underline{\theta}|\underline{\pi})P(\mathcal{D}_{train}|\underline{\theta})}{P(\mathcal{D}_{train}|\pi)} $$

is the a posteriori distribution of parameters $\underline \theta $. As a prior distribution $P(\underline {\theta }|\underline {\pi })$ on the parameters we use a product Dirichlet distribution with hyper parameters $\pi _{m\widetilde {f}}, m \in \widetilde {M}, \widetilde {f} \in \widetilde {F}$ defined as

$$\begin{array}{*{20}l} \pi_{m\widetilde{f}} = \left\{\begin{array}{cl} \alpha, & \widetilde{f} \neq \perp \\ \beta, & \widetilde{f} = \perp \\ \end{array}\color{white} \right\} \end{array} $$

where *α* and *β* are also called pseudo counts.

The parameter estimation is given by

$$\hat{\theta}_{m\widetilde{f}}^{MP} = \frac{N_{m\widetilde{f}} + \pi_{m\widetilde{f}}}{{\sum\nolimits}_{\widetilde{f}' \in \widetilde{F}}~\left(N_{m\widetilde{f}'} + \pi_{m\widetilde{f}'}\right)}. $$

### Fragment losses

Fragment losses can provide additional evidence for a molecular structure as the difference between two *m/z* fragment peaks provides hints about a substructure that was lost but not observed directly by an *m/z* fragment peak (neutral loss). However, we want to include this information in the evaluation of candidates for a given MS/MS spectrum. We define *l*_*nkh*_ to be the *m/z* fragment loss between two different *m/z* fragment peaks *m*_*nk*_ and *m*_*nh*_ from the spectrum $\underline {m}_{n}$, where

$$\begin{array}{*{20}l} l_{nkh} &= m_{nk} - m_{nh}, & m_{nk} > m_{nh}. \end{array} $$

For each pair of assignments of *m/z* fragment peaks to fragment-structures (*m*_*nk*_,*f*_*nck*_) and (*m*_*nh*_,*f*_*nch*_) with *f*_*nch*_ being a genuine substructure of *f*_*nck*_ (*f*_*nck*_≠*f*_*nch*_), we introduce *f*_*nckh*_ as a loss fragment-structure. This fragment-structure is a substructure of *f*_*nck*_, that is generated if all bonds and atoms present in *f*_*nch*_ are removed (*f*_*nckh*_=*f*_*nck*_∖*f*_*nch*_). If *f*_*nckh*_ is connected, we define (*l*_*nkh*_,*f*_*nckh*_) to be an assignment of an *m/z* fragment loss to a fragment-structure.

In analogy to the pairs of *m/z* fragment peaks and fragment-structures (*m*_*nk*_,*f*_*nck*_), we define the domains for the *m/z* fragment losses and loss fragment-structures for the *N* MS/MS training spectra as

$$\begin{array}{*{20}l} \widetilde{L}_{tr} &= \left\{l_{nkh} | n \in 1,\dots N, k=1,\dots K_{n}, h=1,\dots K_{n} \right\} \\\ \widetilde{F}^{L}_{tr} &= \left\{\widetilde{f}_{nckh} | n \in 1,\dots N, c=1,\dots C_{n}, \right.\\ &\quad \ \ \left. {\vphantom{\widetilde{f}_{nckh}}} k=1,\dots K_{n}, h=1,\dots K_{n}\right\} \end{array} $$

for a given training data set

$$\begin{array}{*{20}l} \mathcal{D}^{L}_{train} &= \left((l_{nkh}, f_{nckh}) | n = 1, \dots N, c=1,\dots C_{n},\right. \\ k &= 1, \left. {\vphantom{0_2}} \dots K_{n}, h = 1, \dots K_{n} \right) \end{array} $$

of assignments of *m/z* fragment losses to fragment-structures.

In addition, both domains need to be extended for the additional query MS/MS spectrum $\underline {m}_{q}$

$$\begin{aligned} \widetilde{L} &= \widetilde{L}_{tr} \cup \{l_{qkh} | k=1,\dots K_{q}, h=1,\dots K_{q} \},\\ \widetilde{F}^{L} &= \widetilde{F}^{L}_{tr} \cup\! \left\{\widetilde{f}_{qckh} | c\! = 1,\dots C_{q}, k\! =1,\dots K_{q}, h=1,\dots K_{q} \right\}. \end{aligned} $$

We consider the distribution $P(\underline {\smash {\widetilde {f}}} | \underline {l})$ for assignments of fragment-structures to *m/z* fragment losses with $l \in \widetilde {L}$ and $\widetilde {f} \in \widetilde {F}^{L}$, and denote $\phi ^{L}_{l\widetilde {f}} := P(\underline {\smash {\widetilde {f}}} | \underline {l})$. In analogy to the estimation of the parameters $\theta _{m\widetilde {f}}$, we can now formulate the estimation of $\phi ^{L}_{l\widetilde {f}}$ including a Dirichlet a priori distribution with the additional hyper parameters $\psi _{l\widetilde {f}}$:

$$\begin{array}{*{20}l} \psi_{l\,\widetilde{f}} = \left\{\begin{array}{cl} \alpha^{L}, & \widetilde{f} \neq \perp \\ \beta^{L}, & \widetilde{f} = \perp \\ \end{array}\color{white} \right\} \end{array} $$

This yields the mean posterior estimates

$$\begin{aligned} &\hat{\phi}_{l\,\widetilde{f}}^{MP} = \frac{N^{L}_{l\widetilde{f}} + \psi_{l\widetilde{f}}}{{\sum\nolimits}_{f' \in \widetilde{F}^{L}} \left(N^{L}_{l\widetilde{f}'} + \psi_{l\widetilde{f}'}\right)},\\& \quad \text{ with} \quad N^{L}_{l\widetilde{f}} = \sum\limits_{(l_{t},\widetilde{f_{t}}) \in \mathcal{D}^{L}_{train}} \delta(\widetilde{f}_{t},\widetilde{f})\delta(l_{t},l) \end{aligned} $$

analogous to the parameter estimation for the assignments of *m/z* fragment peaks to fragment-structures, where $N^{L}_{l\widetilde {f}}$ is the absolute frequency of the *m/z* fragment loss and fragment-structure pair $(l,\widetilde {f})$ observed in the training data set $\mathcal {D}^{L}_{train}$.

### Evaluation of the assignments of fragment-structures to *m/z* fragment peaks and losses in MetFrag candidate scoring

To evaluate a given candidate *c* retrieved from a compound database for an MS/MS query spectrum $\underline {m}_{q}$ based on the statistical models, we define a score for both the models of the assignments of *m/z* fragment peaks/losses to fragment-structures. In addition, the MetFrag fragmenter score $S^{c}_{MetFrag}$ as defined in [3] is also integrated in this candidate evaluation. We define the score $S_{Fin}^{c}$ as the final or consensus score for a candidate *c* to be the weighted sum of these three scoring terms

$$\begin{array}{*{20}l} S_{Fin}^{c} &= \omega_{1} \cdot S^{c}_{MetFrag} + \omega_{2} \cdot S^{c}_{Peak} + \omega_{3} \cdot S^{c}_{Loss}\\ \omega_{i} &\ge 0, \sum\limits_{i=1,2,3}\omega_{i} = 1. \end{array} $$

To define $S^{c}_{Peak}$ and $S^{c}_{Loss}$, we first introduce the raw score of a candidate as

$$\begin{array}{*{20}l} S^{c}_{RawPeak} &= \frac{1}{-\log P\left(\underline{\smash{\widetilde{f}}}_{nc}|\underline{m}_{n},\hat{\underline{\theta}}^{MP}\right)} \end{array} $$

using the log likelihood based on the estimated parameters $\underline {\theta }^{MP}$ for the assignment of an *m/z* fragment peak to a fragment-structure $(\underline {m}_{n}, \underline {\smash {f}}_{nc})$ for candidate *c*. With $\underline {\smash {\widetilde {f}}}_{nc} = (\widetilde {f}_{nc1}, \dots, \widetilde {f}_{ncK_{n}})$ and $\underline {m}_{n} = (m_{n1}, \dots, m_{n{K_{n}}})$ the log likelihood decomposes as

$$\begin{array}{*{20}l} \log P\left(\underline{\smash{\widetilde{f}}}_{nc}|\underline{m}_{n},\hat{\underline{\theta}}^{MP}\right) &= \sum\limits_{k=1}^{K_{n}} \log P\left(\widetilde{f}_{nck}|m_{nk},\hat{\underline{\theta}}^{MP}\right). \end{array} $$

Furthermore, the raw score is normalized to the interval [0,1] by

$$\begin{array}{*{20}l} S^{c}_{Peak} &= \frac{S^{c}_{RawPeak}}{\max_{c' \in C_{q}} S^{c'}_{RawPeak}}. \end{array} $$

Using identical ranges for the different scoring terms as for the MetFrag fragmenter score simplifies their integration into the weighted sum of the final score. The score for including the assignments of *m/z* fragment losses to fragment-structures $S^{c}_{Loss}$ is defined in analogy.

### Method evaluation

For the evaluation of the presented approach we used the challenge data set and evaluation procedures of the CASMI 2016 contest. In this contest candidate lists were provided by the organizers along with the spectra to be used by all participants. After the contest, several participants which used statistical learning (e.g. CSI:FID, CSI:IOKR, CFM-ID) coordinated which compounds were used in the training steps to improve the comparability between methods. They exchanged the InChIKeys (InChI: International Chemical Identifier) [15] of the spectra used in training their approaches, although it was not guaranteed that two participants used exactly the same MS/MS spectrum for a compound identified by a common InChIKey if they used different spectral databases. This evaluation is based on 87 of the 208 spectra provided originally in the challenge, as the remaining 121 spectra were removed as they were included in the training data of at least one participant. The results for this subset of the challenge spectra were published in [12] and used here in Table 2 for comparison against MetFrag2.4.5. We used the same set of InChIKeys to obtain the training spectra for this paper. The training data is available from the github repository accompanying the paper.

#### Preparation of the training data set

The training data set includes MS/MS spectra provided by the contest organizers consisting of 312 CASMI training spectra. Participants were allowed to use additional training spectra retrieved from spectral databases e.g. the MassBank of North America (MoNA) [16] and the Global Natural Products Social Molecular Networking (GNPS) [17] spectral library. The InChIKeys of the molecules of these additional spectra were provided by the participants.

We used the provided InChIKeys to retrieve the additional training spectra by querying the MoNA and GNPS spectral databases. For MoNA, retrieved MS/MS spectra from one institution were merged in case more than one spectrum was present for a molecule based on the first block the InChIKey. Thus for one InChIKey several merged spectra can be present in case they originate from different sources. Spectra originating from GNPS spectral database were merged independently of their source. The spectra merging was performed by averaging *m/z* fragment peaks within a specified mass range (given by MS setup of the MS/MS spectra) and retaining the peak of maximum intensity. This resulted in 5 622 spectra (4728 positive and 884 negative) which were used for training. To reduce the spectral complexity only the 40 most abundant (based on intensity) *m/z* peaks in each spectrum were used. The same applies to test spectra used for evaluation.

#### Training of parameters

In the training phase the optimal parameters used to calculate the candidates’ consensus score need to be determined. This parameter set consists of the absolute frequencies $N_{m\widetilde {f}}$ and $N^{L}_{l\widetilde {f}}$ of the assignments of *m/z* fragment peaks and losses to fragment-structures, the hyper parameters *α*,*β*,*α*^*L*^ and *β*^*L*^, and the score weights *ω*_1_,*ω*_2_ and *ω*_3_. The whole training phase described in this paragraph is illustrated in Fig. 2.

Training was separated into two phases where in the first phase the $N_{m\widetilde {f}}$ and $N^{L}_{l\widetilde {f}}$ parameters were determined using only the correct candidate for each training spectrum. Based on these absolute frequencies the optimal hyper parameters and weight scores are determined in the second phase.

If we had used the same data set for the estimation of all parameters, $\mathcal {D}_{train}$ and $\mathcal {D}^{L}_{train}$ would have contained the same pairs of *m/z* fragment peaks/losses and fragment-structures for the correct candidate to be ranked in the second phase. The correct candidate would then be favoured during candidate ranking. This is not representing a realistic case when a query spectrum of an unobserved molecule is processed where we expect also *m/z* fragment peak and loss assignments not previously observed in the optimization phase.

For this reason the complete training data set was split randomly into two disjunct groups of spectra. The splitting was performed by dividing the unique list of InChIKeys (first block) with a ratio of 70:30 and collecting each corresponding spectrum to a group based on the InChIKey of the underlying molecule. The larger group is used in the first phase to calculate the $N_{m\widetilde {f}}$ and $N^{L}_{l\widetilde {f}}$.

In the first phase the correct candidate of each spectrum was processed by MetFrag’s in silico fragmentation. The *m/z* fragment peaks explained by a fragment-structure were corrected to the mass of the molecular formula of the assigned fragment-structure. This is required to be independent of the different mass accuracies of MS/MS spectra acquired under different instrument conditions. Thus the list of assignments of *m/z* fragment peaks/losses to fragment-structures $\mathcal {D}_{train}$ and $\mathcal {D}^{L}_{train}$ contained assignments with the corrected *m/z* values used for the calculation of $N_{m\widetilde {f}}$ and $N^{L}_{l\widetilde {f}}$.

In the second training phase candidates were retrieved from a local PubChem [18] mirror (June 2016) using the monoisotopic mass of the correct candidate of each spectrum and a relative mass deviation dependent on the experimental conditions of the underlying MS measurement. To reduce runtime the correct and at most 500 randomly sampled candidates were processed from the retrieved list of candidates. The rank of the correct candidate was determined and the overall number of Top1 ranks was used as optimization criterion.

For the hyper parameters the optimization was performed by a grid search over an initial domain including a set of all combinations of the values 0.0025, 0.0005 and 0.0001 resulting in a total of 3^4^=81 sets of hyper parameters. If the optimal number of Top1 ranks was located at the border of this hyper parameter domain the search space was extended by increasing or decreasing the parameter by a factor of 5 or 1/5 respectively. This procedure was continued until an optimum was found with an improvement of less than 1% compared to the previous optimum of Top1 ranks. For the score weights a set of 1000 parameter combinations was sampled equally distributed on the simplex. Consensus scores and the rankings of the correct candidates were calculated for all combinations of hyper parameters and weights resulting in initially 81.000 combinations.

Subsequent to this training procedure, the absolute frequencies $N_{m\widetilde {f}}$ and $N^{L}_{l\widetilde {f}}$ were recalculated using the entire training data set to increase the observation domain of assignments of *m/z* fragment peaks/losses to fragment-structures used for the processing of the challenge data set.

#### Fingerprint function

To investigate the effect of the fingerprint function *MolFing* on the results, the complete training phase was performed four times with different fingerprint functions for the same training spectra. For comparison the Lingo- [14], the MACCS- [13], the Circular- [19], and the GraphOnlyFingerprint were used. For calculation of the different fingerprints CDK (version 2.1) [20] implementations were used. The fingerprint with the best training result was selected for the processing of the challenge data set.

#### Processing of the CASMI challenge data set

After the training phase and the selection of the fingerprint function, the in silico fragmentation and scoring was performed for the 87 challenge spectra using the provided candidate lists. Candidates that included non-connected substructures or non-natural isotopes (like deuterium) were discarded from the candidate lists. The candidate ranking was performed after the removal of multiple stereoisomers in compliance with the contest rules and evaluation. Stereoisomers were detected based on the first block of the candidates’ InChIKey representing the molecular skeleton and only the best scoring stereoisomer was regarded for candidate ranking. The results were evaluated and compared on the basis of the average Top1, Top3, and Top10 rankings and the median and mean average rankings of the correct candidate as in [12].

#### Stability of parameter optima and ranking results

Splitting of the training data set for the two phases was performed randomly. As the resulting parameters depend on the splitting, we performed ten independent trials with different splits of the training data. The resulting parameters and their performance on the challenge data set were reported to investigate the effect of randomization.

## Results

### Comparison of different fingerprint functions

**Fig. 3:**
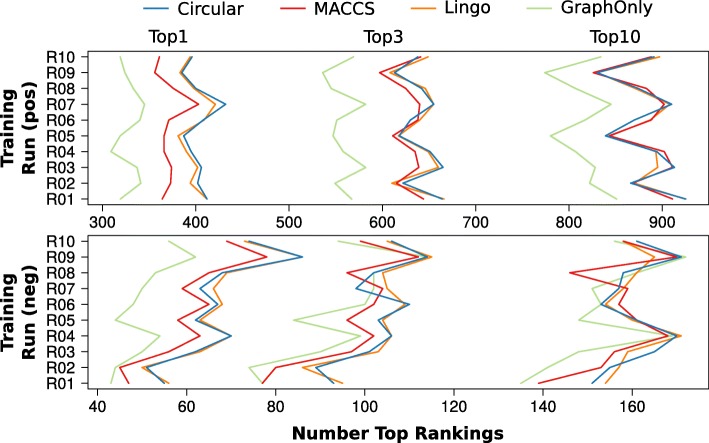
Top rankings of training results. The Top rankings (Top1, Top3, Top10) of the ten training runs are shown for the different fingerprint function. The results are based on the rankings of the correct candidates of the training data used in the second training phase consisting of 1389 to 1471 spectra in positive mode (top) and 255 to 279 spectra in negative mode (bottom)

The ranking results obtained in the training phase on the basis of the different fingerprint functions (*MolFing*) are shown in Fig. 3. The fingerprints used are the Lingo-, MACCS-, Circular-, and GraphOnlyFingerprint. The training results are based on the spectra processed in the second phase during training consisting of 1389 to 1471 spectra in positive and 255 to 279 spectra in negative mode depending on the run and the spectra splitting.

Comparable results are obtained with the Circular- and LingoFingerprint across both ion modes and across the different rankings as shown in Fig. 3 by the similar curve for the Top1, Top3 and Top10 rankings. Similar means of the rankings across the ten runs confirm this observation with 402.3, 639.8, and 881.2 for the mean Top1, Top3 and Top10 rankings using the Circular- and 398.4, 640.0 and 881.9 using the LingoFingerprint. These two fingerprint functions show superior results for the Top1 rankings compared to MACCS with 371.0 and GraphOnly 328.6. For Top3 and Top10 rankings and positive mode the MACCSFingerprint gives comparable results. Top3 and Top10 rankings in negative mode are comparable for all fingerprint functions.

The CircularFingerprint shows with the runs R07 in positive and R09 in negative mode the overall highest number of Top1 rankings with 518 of the 1686 training spectra. Due to this performance the CircularFingerprint is used for subsequent investigations and the evaluation of the challenge data set.

### Randomization of training data sets

**Fig. 4:**
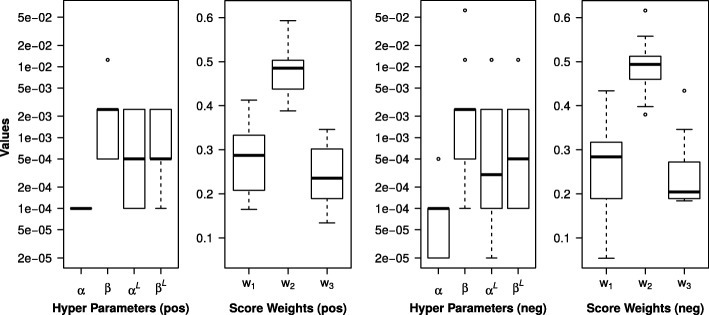
Boxplots of optimal weight and hyper parameters retrieved in the training phase. The parameters were obtained from the ten training runs with randomized splits of the training set and the CircularFingerprint. The rankings results show the optimal weight and hyper parameters for positive and negative mode

In this section we evaluate the impact of the randomization of the training data on parameter optimization. Table 1 shows the optimal parameter sets and the performance achieved on the training data using the CircularFingerprint. The overall ranking results vary across the ten runs for the Top1, Top3 and Top10 numbers in both positive and negative ion mode as expected. Boxplots of the parameter sets are shown in Fig. 4. The variation of optimal hyper parameters as well as weights shows a similar pattern for both positive and negative ion mode where a larger variation can be observed in negative mode. Particularly the pseudo counts for annotated *m/z* fragment peaks show a broader variation with 5e-04 to 2e-05 (*α*) and 1e-03 to 2e-05 (*α*^*L*^) compared to positive mode with 1e-04 as optimum for *α* and an interval of 2e-03 to 1e-04 for *α*^*L*^.

**Table 1:** Ranking results in the training phase based on the CircularFingerprint

Top1	Top3	Top10	Top1 (%)	*α*	*β*	*α* ^*L*^	*β* ^*L*^	*ω*_1_	*ω*_2_	*ω*_3_	# Spectra
**Negative Mode**											
55	93	151	20.8	0.00002	0.00250	0.00050	0.00050	0.268	0.460	0.272	265
51	89	155	19.5	0.00002	0.06250	0.01250	0.00050	0.434	0.380	0.186	261
62	101	165	22.9	0.00050	0.01250	0.00010	0.01250	0.309	0.508	0.184	271
70	106	170	25.8	0.00050	0.00250	0.00002	0.01250	0.317	0.494	0.189	271
62	103	161	23.8	0.00010	0.00010	0.00010	0.00250	0.170	0.616	0.214	260
67	110	153	24.0	0.00010	0.00250	0.00250	0.00010	0.300	0.493	0.207	279
63	98	157	22.9	0.00010	0.00050	0.00010	0.00050	0.054	0.512	0.434	275
68	102	158	25.0	0.00002	0.00250	0.00250	0.00250	0.240	0.558	0.202	272
86	114	171	31.2*	0.00010	0.00250	0.00250	0.00010	0.413	0.398	0.189	276
74	106	161	29.0	0.00010	0.00010	0.00002	0.00010	0.189	0.465	0.346	255
**Positive Mode**											
412	664	925	28.0	0.00010	0.00250	0.00010	0.00250	0.333	0.438	0.229	1471
402	622	866	28.2	0.00010	0.00050	0.00010	0.00250	0.208	0.483	0.309	1426
406	665	913	29.0	0.00010	0.01250	0.00250	0.00250	0.333	0.438	0.229	1399
395	651	894	27.6	0.00010	0.00250	0.00250	0.00250	0.309	0.503	0.188	1432
387	618	839	27.4	0.00010	0.00250	0.00050	0.00050	0.413	0.398	0.189	1413
408	630	870	28.6	0.00010	0.00050	0.00050	0.00050	0.165	0.584	0.251	1428
432	655	910	30.6*	0.00010	0.01250	0.00250	0.00050	0.378	0.488	0.134	1410
400	642	874	28.2	0.00010	0.00250	0.00250	0.00050	0.210	0.488	0.302	1420
385	613	830	27.7	0.00010	0.00250	0.00010	0.00010	0.266	0.388	0.346	1389
396	638	891	27.7	0.00010	0.00050	0.00050	0.00010	0.165	0.593	0.242	1428

The optimization of the parameters was performed on the training data set with ten different random splits of the MS/MS training spectra to be used for first and second training phase. Optimization was performed separately for positive and negative mode. *Runs with the best results based on the relative correct Top1 rankings (neg: R09, pos: R07)

**Table 2:** Results for the 87 MS/MS test spectra from the CASMI 2016 Challenge taken from Table 7 in [12] augmented with the results of the proposed approach (MetFrag 2.4.5). For the participants of the challenge the best result is given

	MetFrag 2.4.5	MetFrag 2.3	CFM-ID	CSI:IOKR	CSI:FID	MS-Finder	MAGMa
Top 1 Pos.	9	1	9	10	13	3	2
Top 1 Neg.	12	4	6	6	−^∗^	7	4
Top 1	21	5	15	16	13 ^∗^	10	6
Top 3	38	16	24	26	23 ^∗^	25	16
Top 10	55	39	40	46	32 ^∗^	38	35
Mean rank	34.6	68.4	64.1	97.9	41.5 ^∗^	28.7	76.8
Med. rank	5	14.5	12.5	10	3 ^∗^	17.5	23.5

^*^CSI:FID did not submit results for negative mode spectra

The largest of the weights combining the three scores is *ω*_2_ which gives the score $S^{c}_{Peak}$ the largest influence in the overall assessment. The median of *ω*_2_ is 0.4855 in positive and 0.4935 in negative mode. The impact of the original MetFrag score $S^{c}_{MetFrag}$ and $S^{c}_{Loss}$ are distinctively lower and comparable to each other. The weight *ω*_1_ for the MetFrag score has a median of 0.2875 in positive and 0.2840 in negative mode. The weights for *ω*_3_ are 0.2355 respectively 0.2045.

In the following we analyze the robustness and the homogeneity of the results on the challenge data set with regard to varying parameters across the parameter space evaluted during optimization. This also helped to obtain a better explanation on the deviation of optimized parameters. Specifically we compare the distribution of the Top1 rankings considering (i) the ten optimal parameter sets from the ten randomizations, (ii) the parameter sets within the convex hull constituted by these ten optimal parameter sets in the six dimensional parameter space, and (iii) the complete parameter space evaluated during training of the parameters. The convex hull over the ten optimal parameter sets was calculated using the six degrees of freedom (*α*,*β*,*α*^*L*^,*β*^*L*^,*ω*_1_,*ω*_2_) from the seven parameters with the Python *Numpy* package.

**Fig. 5:**
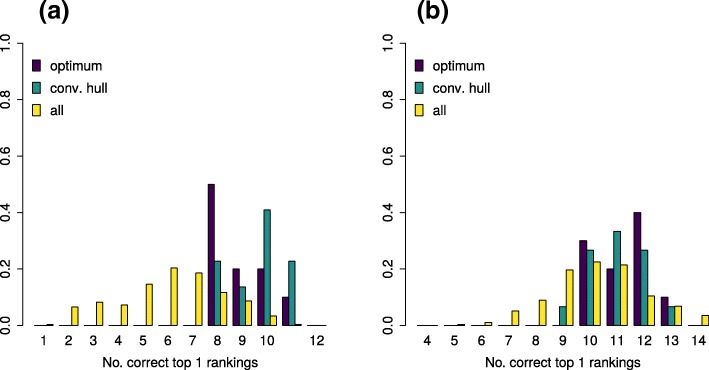
Distribution of Top1 rankings on the challenge data set. The collection of barcharts show the Top1 rankings retrieved using the CircularFingerprint for selected parameter sets. Yellow bars show the normalized Top1 counts for all parameter sets used in the training phase. The green bars show the normalized rankings for all parameter sets within the convex hull spanned by the ten optimal parameter sets retrieved from the ten randomized training runs. The violet bars show the normalized counts from these optimal parameter sets. **a** Positive mode **b** Negative mode

Figure 5 shows in yellow the distribution of the Top1 rankings of the CASMI challenge data set for the complete parameter space. Top1 ranking vary from 1 to 12 for the positive and from 4 to 14 for the negative challenge spectra, where the maximum of the distributions are six and ten for positive and negative mode, respectively. If parameter sets are restricted to the convex hull the distribution is clearly shifted to better performance, where Top1 rankings vary between 8 to 11 for positive and 10 to 13 for negative mode. This range of Top1 rankings is almost identical to the one resulting from the ten optimal parameter sets. The only exception are nine Top1 rankings for parameter sets within the convex hull in negative mode. In positive mode about 76% of the investigated parameters show worse results than achieved by the parameters contained in the convex hull. For negative mode this proportion is reduced to around 15% which can again be explained by the smaller number of available training data.

For the subsequent comparison to other methods on the challenge data set we use the parameter sets resulting in the best relative Top1 ranking performance in the training phase. The corresponding runs are highlighted in Table 1 and are R07 for positive and R09 in negative mode.

### Comparison with MetFrag2.3

The main goal of the integration of the proposed approach into MetFrag was to improve the candidate ranking augmenting the fragmenter score with statistical scores. The MetFrag versions 2.3 and 2.4.5 use exactly the same in silico fragmentation approach. MetFrag2.4.5 scoring was extended with the statistical scoring terms which make the difference in the comparison of both version. The results of MetFrag version 2.4.5 show a drastic improvement of the rankings for the CASMI challenge data compared to its older version 2.3 with regard to all performance measures as given in the first two columns of Table 2. The correct Top1 rankings show a more than four fold increase from 5 to 21 Top1 rankings. The improvement is especially distinct for positive mode with 9 Top1 rankings where MetFrag2.3 resulted in one single query correctly ranked at first position. The number of Top1 hits in negative mode is also increased three fold from 4 to 12. The improvement is also illustrated by the reduced mean and median ranks. Where the mean rank halved to 34.6 the median rank was even reduced by two third to 5. All three scores contribute substantially to these improvements and Top1 rankings vary smoothly with the weight scores (see Additional file 1: Figure S1).

### Comparison with other CASMI participants

The MetFrag2.4.5 results were compared to the results obtained by all other participants of CASMI 2016, i.e., CFM_retrain, CSI_IOKR_AR, and CSI:FID_leaveout (abbreviated by CFM-ID, CSI:IOKR, and CSI:FID), MS-Finder and MAGMa. Table 2 shows the original data from Table 7 of [12] with the ranking results for the 87 Challenge MS/MS spectra. The additional MetFrag2.4.5 column summarizes the results achieved using the new MetFrag statistical scoring terms.

**Fig. 6:**
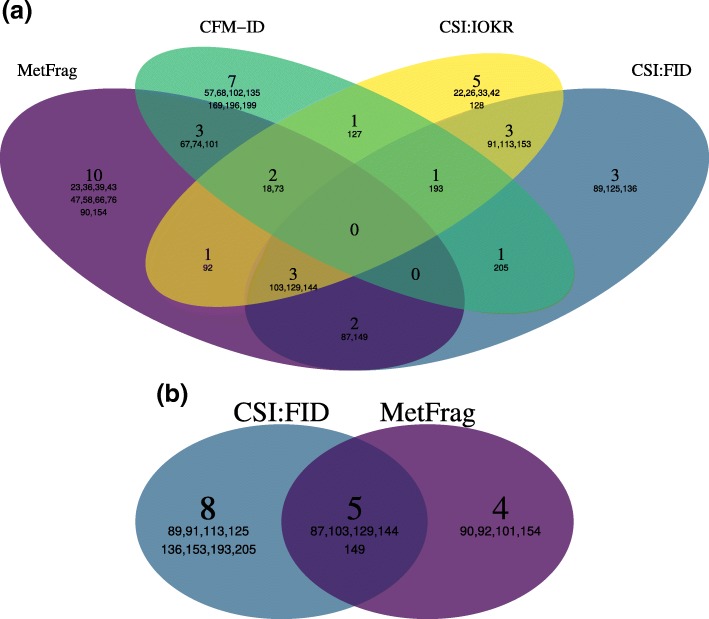
Overlap of the correctly identified Top1 spectra of the challenge data set for selected participants. The Venn diagram (**a**) includes the four tools using statistical approaches (MetFrag2.4.5, CFM-ID, CSI:IOKR, CSI:FID) and shows the overlap of correcly identified challenges out of the 87 spectra (positive and negative mode). The diagram (**b**) shows the overlap of CSI:FID and MetFrag2.4.5 for the positive mode challenges. The large numbers indicate the amount of common challenges and the numbers listed underneath their challenge IDs

In positive mode, MetFrag2.4.5 obtains nine Top1 rankings and shows a similar performace as CFM-ID (9) and CSI:IOKR (10). CSI:FID (13) outperforms all other approaches with regard to Top1 rankings in positive mode, however did not submit results for negative mode spectra. Figure 6b shows the overlap of the Top1 ranked challenges in positive mode for MetFrag2.4.5 and CSI:FID. There are only five challenges ranked first by both tools and thus a large degree of divergence between the correct predictions.

For the negative mode spectra MetFrag2.4.5 considerably outperformed all participants with 12 Top1 rankings. These are five more queries than MS–Finder could rank in first position and even twice as many than the other statistical approaches CFM-ID and CSI:IOKR.

Considering the complete test data set MetFrag2.4.5 outperforms all participants with regard to Top1, Top3, and Top10 rankings including the declared winner of the contest CSI:IOKR (Top1: 21, Top3: 38, Top10: 55 vs. Top1: 16, Top3: 26, Top10: 46). The improved results are also confirmed by the smaller median and mean rankings of 5 and 34.6 compared to 10 and 97.9. We note that considering the median, CSI:FID shows a better performance than MetFrag2.4.5, however did only submit results for positive mode.

Figure 6a shows the overlap of correctly identified Top1 challenges of the participants which use statistical approaches. Interestingly, there is a relatively large number of challenges that are identified by only one of the approaches. With 10 challenges MetFrag2.4.5 shows the highest amount of unique queries ranked correctly in first place, which is predominantly caused by the eight Top1 negative mode challenges.

## Discussion

The results obtained by the combination of MetFrag’s in silico fragmentation approach and statistical fragment annotation learning have shown an overall improvement of the ranking results of the relevant CASMI 2016 test set. Different fingerprint functions have been tested to avoid the expensive graph isomorphism problem to find matching fragments. The training phase revealed a dependency between the number of correct top hits and the fingerprint used. While MACCS- and especially Lingo- and the CircularFingerprint showed the best and also comparable results, the GraphOnlyFingerprint showed a significantly lower number of correct top rankings on the training set. We attribute the inferior performance of the GraphOnlyFingerprint primarily to the lack of representing bond orders and hence encoding less chemical information than all other fingerprint types evaluated. Due to the best performance in the training phase the CircularFingerprint was selected for further investigation on the test set.

Ten different hyper and weight parameter sets resulting from optimization with ten randomized splits of the training data were used to investigate the robustness and the distribution of these parameters accross the different training sets. While the optima of the seven parameters varied slightly between the different splits, the parameter sets still showed a clear trend across all ten runs. Especially the effect of the $S^{c}_{Peak}$ score weight *ω*_2_ was predominantly higher compared to *ω*_1_ and *ω*_3_ for both positive and negative ion mode. The assumption that the observed parameter variation is an indication for a relatively broad and homogenious parameter optimum was confirmed by the investigation of the ranking results retrieved using parameters located in the convex hull spanned by the ten optima. These distributions also indicate a high robustness of the performance with varying parameter sets across these parameter optima.

An important outcome of this study is the significant improvement of the ranking results retrieved adding the presented Bayesian approach to MetFrag’s native in silico fragment annotation. While the improvement gain for the Top3 and Top10 rankings are less pronounced, this comparison impressively demonstrates the benefit including statistical approaches for MS based compound identification. This corresponds to the outcome of CASMI 2016 where a comparison of different statistical and non-statistical approaches was made [12].

The proposed Bayesian approach follows a different mechanism than the existing statistical compound identification methods predicting molecular fingerprints (CSI:FingerID, CSI:IOKR) or MS/MS spectra (CFM-ID). The comparison of the different approaches on the CASMI 2016 test set used in this study shows on the one hand that the presented approach compares well to the existing ones and on the other hand that a relatively large number of challenges are identified by only one of the approaches (Fig. 6a). From the latter finding it may be concluded that there are different preferences for certain types of spectra of the CASMI 2016 contest. The comparison also revealed that for MetFrag2.4.5 the performance is comparable between positive and negative mode (9 vs. 12). CSI:IOKR shows lower performance ranking result for the negative mode spectra compared to positive mode (6 vs. 10). We assume the combination of in silico fragmentation and statistical scoring has a positive effect in case only limited training data is available. Only a small fraction of negative mode training data was available for this contest and resulted in generally worse results of the statistical approaches in negative mode.

## Conclusions

In this work new statistical scoring terms are introduced to MetFrag. This model assesses the assignments of *m/z* fragment peaks/losses to fragment-structures derived from in silico fragmentation of a candidate and assumes independence of the individual assignments. The model parameters are estimated using the mean posterior approach. Hyper parameters of the statistical model as well as score weights are optimized by a grid search. The performance is evalutated on a subset of the CASMI 2016 contest challenge spectra for which the spectrum was not among the training data set of any participant. The results show that with the integration of the two new statistical scoring terms MetFrag could be improved four fold regarding the number of Top1 rankings. In addition it showed a better performance than the declared winner of the contest CSI:IOKR regarding the number of correctly ranked Top1, Top3 and Top10 candidates. The new scoring terms are now available in the command line tool (version 2.4.5) as AutomatedPeakFingerprintAnnotationScore and AutomatedLossFingerprintAnnotationScore and also in the web interface (https://msbi.ipb-halle.de/MetFrag) as “Statistical Scoring” trained on extended data set than used in this work. The additional scoring terms complement current scoring terms based on experimental data and can also be combined with additional meta information if available as described in [3].

We also want to stress that once the method is trained on spectra in the training phase, it can be applied and used for annotation on any data set. The data set can vary whereas the training data set is fixed once the method was trained, which is similar to all other machine learning and statistical methods mentioned in this work.

## Additional files

Additional file 1 Figure S1 - Weight Parameter Scan for the test dataset. (PDF 767 kb)

Additional file 2 Figure S2 - Maximum spectral similarities. (PDF 196 kb)

Additional file 3 Figure S3 - Rankings of the correct candidates (test) vs. max. spectral similarity. (PDF 204 kb)

Additional file 4 Table S1 - Notation summary. (PDF 109 kb)

Additional file 5 Table S2 - Notation summary (Scores). (PDF 70.4 kb)

## Data Availability

The *m/z* peak and candidate lists used in this study is available on the official CASMI website, http://www.casmi-contest.org/2016/index.shtml. A complete list of the used MassBank and GNPS training spectra and the ranking data sets generated during the current study are available on GitHub, https://github.com/c-ruttkies/metfrag_statistical_annotation. Further information on how to use the new scoring terms with the commandline version of MetFrag can be found on the project website http://ipb-halle.github.io/MetFrag/projects/metfragcl. The source code is published on GitHub (https://github.com/ipb-halle/MetFragRelaunched (branch: feature/statistical\_scoring)).

## Abbreviations

CASMI: Critical assessment of small molecule identification
CSI: FID: CSI:fingerID
InChI: International chemical identifier
MP: Mean posterior
MS/MS: Tandem mass spectrometry
m/z: Mass-to-charge ratio
mzabs: Absolute mass deviation
mzppm: Relative mass deviation
SVM: Support vector machines
