# Supplementary material for: Improving MetFrag with statistical learning of fragment annotations
Source: BMC Bioinformatics. 2019 Jul 5;20:376. doi: 10.1186/s12859-019-2954-7 (PMC6612146; doi:10.1186/s12859-019-2954-7)

Figure S1: **Weight Parameter Scan for the test dataset.** The plots show the distribution of the number of Top1 rankings for the 1000 combinations of the weight parameters ω_1 (MetFrag), ω_2 (Peak), ω_3 (Loss) for (a) the negative and (b) the positive mode test dataset. The shown Top1 rankings result for the optimum α, β, α^L, β^L parameter set for negative and positive mode as highlighted in Table 7. The circles mark specific quantiles (0.1%, 25%, 50%, 75%, 100%) and their Top1 ranking values.

(a) Negative mode

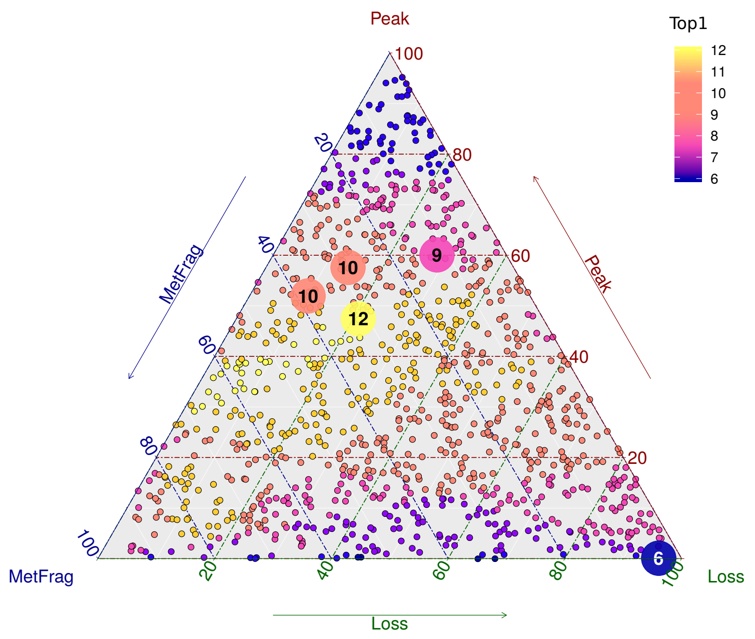

(b) Positive mode

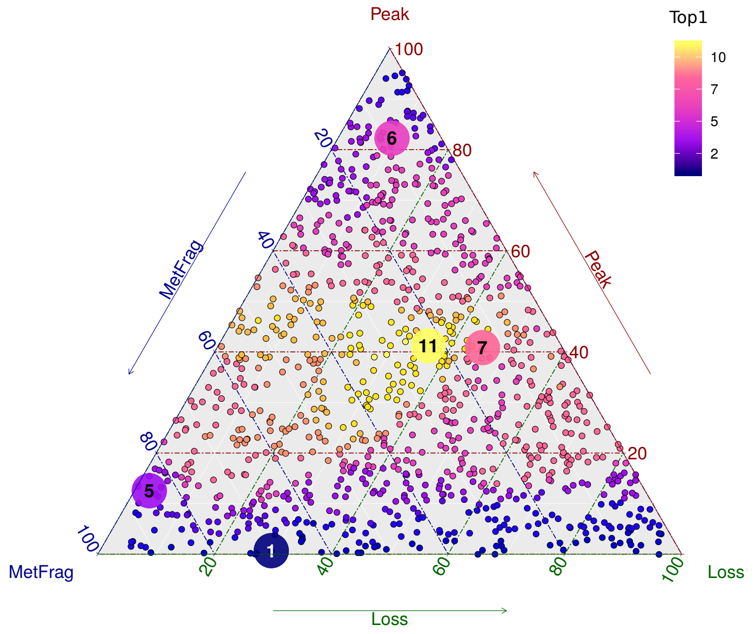

Supplement: Supplementary file 1 — Figure S1 - Weight Parameter Scan for the test dataset. (PDF 767 kb) [file 12859_2019_2954_MOESM1_ESM.pdf]
