# Supplementary material for: Improving MetFrag with statistical learning of fragment annotations
Source: BMC Bioinformatics. 2019 Jul 5;20:376. doi: 10.1186/s12859-019-2954-7 (PMC6612146; doi:10.1186/s12859-019-2954-7)

Figure S2: **Maximum spectral similarities.** Maximum spectral similarities found in the training specta set (excluding CASMI 2016 training spectra) for each challenge (test) spectrum. Top: 44 challenge spectra in positive mode. Bottom: 43 challenge spectra in negative mode. The figures indicate the similarity of training and test spectra used.

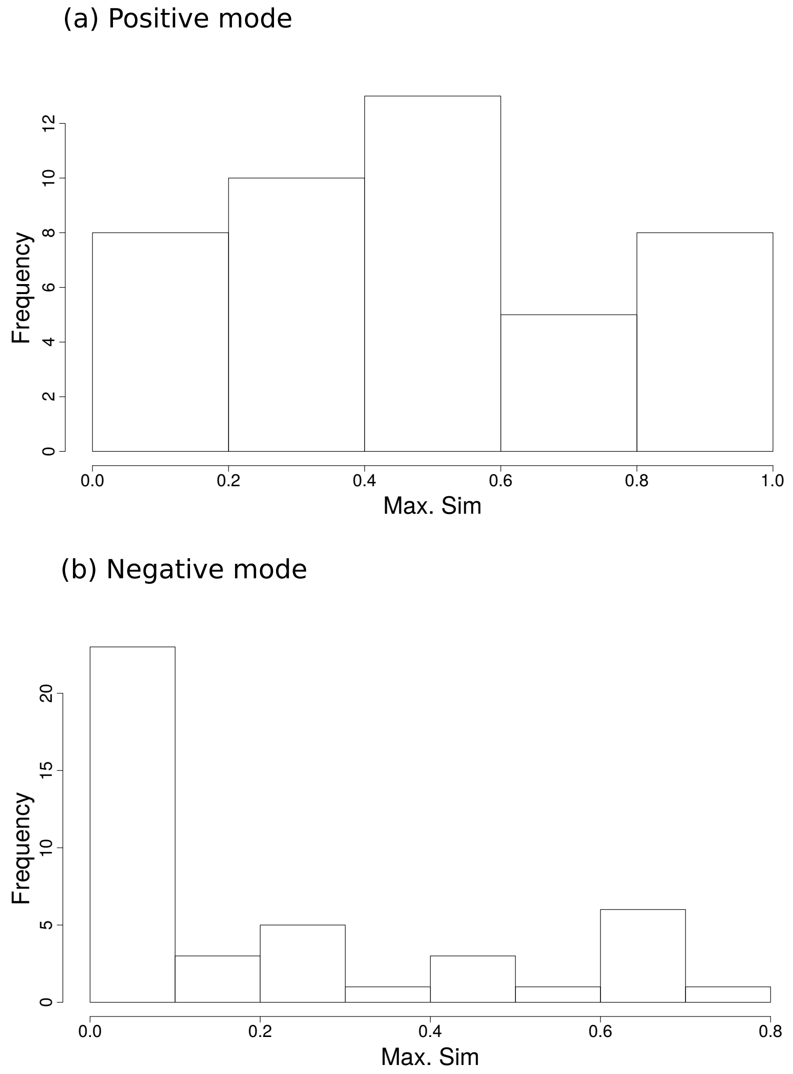

Supplement: Supplementary file 2 — Figure S2 - Maximum spectral similarities. (PDF 196 kb) [file 12859_2019_2954_MOESM2_ESM.pdf]
