# Supplementary material for: Improving MetFrag with statistical learning of fragment annotations
Source: BMC Bioinformatics. 2019 Jul 5;20:376. doi: 10.1186/s12859-019-2954-7 (PMC6612146; doi:10.1186/s12859-019-2954-7)

Figure S3: **Rankings of the correct candidates (test) vs. max. spectral similarity.** Top: Positive challenge spectra (44). Bottom: Negative challenge spectra (43). The figures indicate the correlation between the maximum spectral similarity found in the training spectral set for each test spectrum and the corresponding rank of the correct candidate. The pearson correlation coefficients of -0.21 in positive and -0.03 in negative mode show only a small and even no correlation. Besides a high spectral similarity also a high similarity of the molecular structures of the training and test spectrum is relevant for a good rank in the test phase.

(a) Positive mode

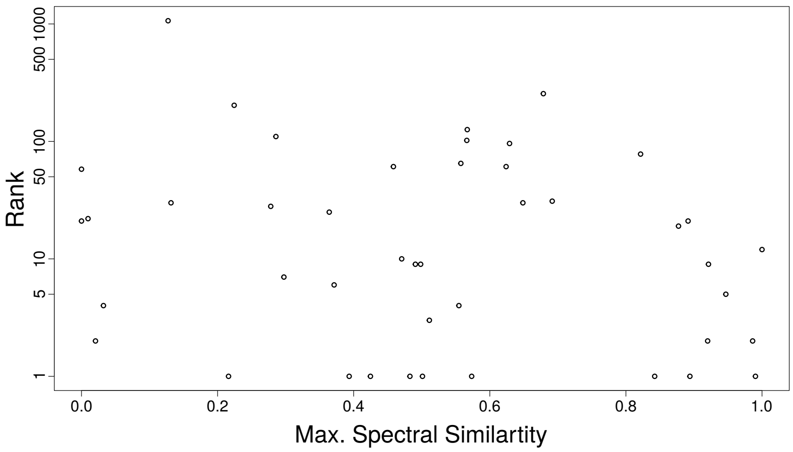

(b) Negative mode

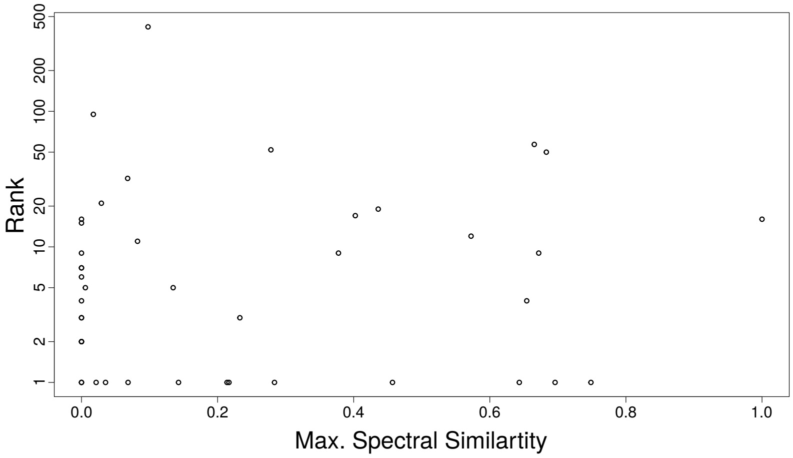

Supplement: Supplementary file 3 — Figure S3 - Rankings of the correct candidates (test) vs. max. spectral similarity. (PDF 204 kb) [file 12859_2019_2954_MOESM3_ESM.pdf]
