# Supplementary material for: Improving MetFrag with statistical learning of fragment annotations
Source: BMC Bioinformatics. 2019 Jul 5;20:376. doi: 10.1186/s12859-019-2954-7 (PMC6612146; doi:10.1186/s12859-019-2954-7)
Supplement: Supplementary file 4 — Table S1 - Notation summary. (PDF 109 kb) [file 12859_2019_2954_MOESM4_ESM.pdf]

Table S1: Notation summary

$\underline{m}$	complete spectral dataset consisting of N MS/MS spectra
$\underline{m}_n$	the n-th MS/MS spectrum of the spectra dataset ($\underline{m}$)
K_n	number of m/z fragment peaks in the n-th MS/MS spectrum
m_{nk}	the k-th m/z fragment peak of the n-th MS/MS spectrum
$\underline{c}_n$	candidate list/set for the n-th MS/MS spectrum
C_n	size/length of candidate list/set $\underline{c}_n$
c_{nc}	$c-th$ candidate of candidate list/set $\underline{c}_n$
f_{nck}	fragment-structure (of candidate c_{nc}) assigned to m/z fragment peak m_{nk}
$(\underline{m}_n, \underline{f}_{nc})$	peak fragment assignment of candidate c_{nc} to MS/MS spectrum $\underline{m}_n$
$\perp$	placeholder indicating a non-annotated annotated m/z fragment peak
$\widetilde{f_k}$	fingerprint of fragment-structure f_k
$\widetilde{M}_{tr}$	reduced m/z fragment peak domain (for training MS/MS spectra)
$\widetilde{F}_{tr}$	reduced fingerprint domain (for training MS/MS spectra)
$\mathcal{D}_{train}$	list of single peak fragment assignments
$\underline{m}_q$	query MS/MS spectrum
m_{qk}	the k-th m/z fragment peak of the query MS/MS spectrum
f_{qck}	fragment-structure (of candidate c_{qc}) assigned to m/z fragment peak m_{qk}
K_q	number of m/z fragment peaks in the query MS/MS spectrum
C_q	candidate list/set of query MS/MS spectrum $\underline{m}_1$
$\widetilde{M}$	reduced m/z fragment peak domain (for training MS/MS spectra + query MS/MS spectrum)
$\widetilde{F}$	reduced fingerprint domain (for training MS/MS spectra + query MS/MS spectrum)
$\theta_{m\widetilde{f}}$	probability of fragment fingerprint $\widetilde{f}$ given m/z fragment peak m
$\theta^{ML}_{m\widetilde{f}}$	maximum likelihood estimator for $\theta_{m\widetilde{f}}$
$N_{m\widetilde{f}}$	absolute frequency of observing fragment fingerprint $\widetilde{f}$ and m/z fragment peak m
$N_{m\perp}$	absolute frequency of observing a non-annotated m/z fragment peak m
$\pi_{m\widetilde{f}}$	hyper parameter for prior distribution
α, β	pseudo counts for prior distribution
$\theta^{ML}_{m\widetilde{f}}$	mean posterior estimator for $\theta_{m\widetilde{f}}$
l_{nkh}	m/z fragment loss between m/z fragment peaks m_{nk} and m_{nh}
f_{nchk}	fragment-substructure of f_{nck} excluding f_{nch} assigned to l_{nkh}
$\widetilde{L}_{tr}$	reduced m/z fragment loss domain (for training MS/MS spectra)
$\widetilde{F}^{L}_{tr}$	reduced loss fingerprint domain (for training MS/MS spectra)
$\mathcal{D}^{L}_{train}$	list of single loss fragment assignments
$\widetilde{M}^{L}$	reduced m/z fragment loss domain (for training MS/MS spectra + query MS/MS spectrum)
$\widetilde{F}^{L}$	reduced loss fingerprint domain (for training MS/MS spectra + query MS/MS spectrum)
$\psi_{m\widetilde{f}}$	hyper parameter for prior distribution of fragment loss model
α^{L}, β^{L}	pseudo counts for prior distribution of fragment loss model
$\phi_{l\widetilde{f}}$	probability of loss fragment fingerprint $\widetilde{f}$ given m/z fragment peak m
$\phi^{ML}_{l\widetilde{f}}$	maximum likelihood estimator for $\psi_{l\widetilde{f}}$
$N^{L}_{l\widetilde{f}}$	absolute frequency of observing fragment loss fingerprint $\widetilde{f}$ and m/z fragment loss m
$S^{c}_{MetFrag}$	MetFrag score of a candidate c
S^{c}_{Peak}	statistical score evaluating fragment - m/z peak assignments of a candidate c
S^{c}_{Loss}	statistical score evaluating loss fragment - m/z loss assignments of a candidate c
$S^{c}_{RawPeak}$	non-normalized statistical score evaluating fragment - m/z peak assignments of a candidate c
ω_1, ω_2, ω_3	single score weights
S^{c}_{Fin}	final/consensus score of a candidate c
