# Supplementary material for: Improving MetFrag with statistical learning of fragment annotations
Source: BMC Bioinformatics. 2019 Jul 5;20:376. doi: 10.1186/s12859-019-2954-7 (PMC6612146; doi:10.1186/s12859-019-2954-7)
Supplement: Supplementary file 5 — Table S2 - Notation summary (Scores). (PDF 70.4 kb) [file 12859_2019_2954_MOESM5_ESM.pdf]

Table S2: Notation summary (Scores)

$S^c_{MetFrag}$	MetFrag score of a candidate c
S^c_{Peak}	statistical score evaluating fragment - m/z peak assignments of a candidate c
S^c_{Loss}	statistical score evaluating loss fragment - m/z loss assignments of a candidate c
$S^c_{RawPeak}$	non-normalized statistical score evaluating fragment - m/z peak assignments of a candidate c
ω_1, ω_2, ω_3	score weights
S^c_{Fin}	final/consensus score of a candidate c
